# How training quality, trainer competence, and satisfaction with training affect vocational identification of apprentices in vocational education programs

**DOI:** 10.3389/fpsyg.2024.1200279

**Published:** 2024-03-22

**Authors:** Eveline Wuttke, Karin Heinrichs, Kristina Koegler, Andreas Just

**Affiliations:** ^1^Chair of Economics and Business Education, Goethe University Frankfurt, Frankfurt, Germany; ^2^Forum of Research on Vocational Education and Training, Institute of Vocational Education, University of Teacher Education Upper Austria, Linz, Austria; ^3^Department of Vocational, Business and Technical Education, Institute for Educational Science, University of Stuttgart, Stuttgart, Germany

**Keywords:** social identity theory, vocational identification, organizational identification, career identification, job satisfaction, training quality, trainer competence

## Abstract

Vocational identification means being identified with an organization and with one’s career. Both are key objectives of vocational education and training (VET) programs and advantageous for employees and employers. For employees, vocational identification is often associated with positive work-related emotions and job satisfaction; for employers, workers’ identification with the organization and the career enhances their performance and reduces turnover. Thus, investment in employees’ professional development that has the potential to support vocational identification is advantageous for all involved. In light of current demographic changes and a decreasing demand for full-time work, which are leading to a shortage of skilled workers and lower enrolment in apprenticeship programs, it is essential to bind young talents to companies at an early stage and avoid resignations during or after training. Findings from various empirical studies confirm that those who identify with their chosen career and the organization for which they work are more satisfied, think less about quitting, and perform better. Little empirical research has been conducted on how apprentices in VET programs identify with their career or organization or the extent to which such identification enhances their job satisfaction. In this study, we therefore investigate factors that influence apprentices’ identification with their career and organization, in particular, the effects of training quality and trainer competence. Our results indicate that apprentices identify strongly with their career and with the organization where they are doing their training and are mostly satisfied with the quality of their training. Structural equation modeling reveals the relevance of career choice, training quality, and job satisfaction for identification with an organization and (less) with a career. The learning and working conditions in the organization, and more specifically, the variety of tasks offered to the apprentices and the trainer’s pedagogical aptitude explain satisfaction with the training and career identification; the trainer’s presence and the apprentices’ satisfaction with training explain, to some degree, variance in organizational identification.

## 1 Introduction

Vocational identification means being identified with an organization and with one’s career. Both are key objectives of vocational education and training (VET) programs and advantageous for employees and employers. For employees, such identification is often associated with positive work-related emotions and job satisfaction; for employers, workers’ identification with an organization and with their career enhances their performance and reduces turnover (cf. Van Dick et al., 2004; Haslam and Van Dick, 2011; van Dick, 2017). Thus, investing in professional development and training which has the potential to support vocational identification is advantageous for all involved.

The construct of vocational identification is in line with the assumptions of social identification theory (Tajfel and Turner, 1979, 1986, for more detail see chapter 2). Social identification is defined as the extent to which the norms, values, and attitudes of a social unit (an organization or people having a similar career) are adopted, that is, the individual defines himself or herself in terms of these characteristics and goals (Mael and Tetrick, 1992). Vocational identification is becoming increasingly important due to recent changes in labor markets: No longer are occupations chosen at a young age and pursued over a lifetime within the same organization where initial training took place, and permanent, full-time employment with one company over a lifetime is becoming a relic of the Industrial Age (Statistische Ämter des Bundes und der Länder, 2012). In 2013, approximately 30% of jobs in Germany requiring skilled workers were difficult to fill (Autorengruppe Bildungsberichterstattung, 2018). Between 2011 and 2022 the number of job vacancies requiring skilled workers across the European Union doubled from 1.6 to 3.2% (Eurostat, 2023), and this problem is expected to become even more acute by 2035 (BIBB, 2019). The central reasons for job vacancies in most developed countries are related to demographic changes such as the retirement of baby boomers and a shrinking population (Mason et al., 2022). Furthermore, tendencies toward greater flexibility in the world of work are increasingly weakening the stability and continuity of careers and leading to more individualization in work life (Vondracek et al., 2010; Kirchknopf and Kögler, 2020).

In addition to the developments outlined above, increasing enrollment in academic programs does not necessarily contribute to a supply of skilled workers and may lead to fewer qualified applicants being available for apprenticeships. Nevertheless, dual vocational training combining theoretical and practical training continues to be in great demand in German-speaking countries, with more than half of an age cohort still seeking vocational education in Germany and more than one third of 15-year-olds doing the same in Austria (Dornmayr, 2021). In Germany, the number of new apprenticeship contracts signed in 2021 rose by 1.2% from the previous year but nevertheless remained well below the figure for 2019, the year before the pandemic (Berufsbildungsbericht, 2023). In Austria, similar developments can be seen (Dornmayr and Löffler, 2022). Furthermore, entering VET programs and remaining in the chosen occupation is not always successful: every year, approximately 25% of training contracts are terminated prematurely,^1^ whereby the entry phase seems to be particularly critical. After the entry phase, the quality of training and the training conditions are decisive for staying in the training program (BIBB, 2022; Böhn and Deutscher, 2022). Overall, retaining new recruits is a challenge, as trainees do not necessarily remain in their first choice of occupation or in the organization where they receive training (Schönfeld et al., 2020, p. 16).

To sum up, findings from various empirical studies confirm that those employees who show higher vocational identification are more satisfied, think less about quitting, and perform better (van Dick, 2017). The developments described above illustrate the importance of apprentices’ vocational identification which - in addition to developing professional skills - is a key objective of all VET programs (Heinrichs et al., 2022). However, little empirical research has been conducted on vocational identification of apprentices in VET programs and the extent to which this enhances their job satisfaction.

Our study is based on social identity theory (for more details on the construct, see Heinrichs et al., 2022) and situated in the context of VET. We specifically focus on two aspects of vocational identification, namely organizational identification and career identification. In our study we investigate factors that are assumed to affect apprentices’ vocational identification, in particular, training quality and trainer competence, and how they relate to vocational identification and job satisfaction.^2^

## 2 Theoretical framework and state of research

### 2.1 Social identity theory and related constructs

Various theoretical approaches (e.g., social identity theory, personal identity, metacognition of vocationality; Heinrichs et al., 2022) can be considered to investigate (1) whether and how vocational trainees identify with their career and the organization where they are doing their training, (2) how satisfied trainees are with their work and the quality of their training, and (3) whether and how this is ultimately reflected in their intention to continue working in their field and organization (Böhn and Deutscher, 2022).

In this paper the focus is on social (vocational) identification in the context of vocational education and training. We follow the assumptions of social identification theory (Tajfel and Turner, 1979, 1986). Social identification is defined as the extent to which the norms, values, and attitudes of a social unit (e.g., people working in the same company or people having a similar career) are adopted, that is, the individual defines himself or herself in terms of these characteristics and goals (Mael and Tetrick, 1992). Through social identification, the identity boundary between the individual and the corresponding group becomes blurred. Differences from and similarities to other group members contribute to the development of social identity, which then result in self-definition. One’s own identity and self-image are thus partly derived from belonging to the group in question (Hogg and Terry, 2001). In the literature, a further distinction is made between dimensions (e.g., van Dick, 2017) and foci of identification (e.g., Wagner and Ward, 1993; van Dick et al., 2004, 2005; van Dick, 2017). However, this differentiation will not be explored in depth here, as it is irrelevant for this study.

In this paper we investigate two facets of social identification within the context of VET: identification with a career and identification with an organization. Both relate to how people define themselves at least in part through their memberships in groups in the work context (e.g., the group of people working in the same organization or those having a similar career). People who identify with their career or organization no longer act in these groups merely as individuals and according to their own convictions, but rather as members of the group to which they feel they belong (Schuh et al., 2020, 3; van Dick, 2017, 18). In research, the concept of *organizational identification*, that is, attachment to an organization, has received significant attention. It describes an employee’s feeling that his or her personality merges with the “personality” of the organization and is largely defined by membership in this group (Van Dick, 2017). This leads to employees perceiving themselves as a unit with the organization and seeing its successes and failures as their own (Mael and Ashforth, 1992). They often internalize values or goals attributed to the organization as their own (Mael and Ashforth, 1995) and integrate them with their self-concept (Hassan, 2012). *Career identification* as another facet of social identification (Mael and Ashforth, 1992; Mael and Ashforth, 1995) emphasizes that the perception of oneself is fed particularly (though not exclusively) by interactions and comparisons in social, work-related contexts with people who have a similar/the same career (Tajfel, 1978; Ellemers et al., 2002; Kirpal, 2004). Career identification is not tied to a specific organization but rather refers to the occupational group to which a person belongs by practicing a particular occupation (Mael and Ashforth, 1992). Through career identification, the individual and the occupational group “merge” with each other so that an individual ultimately identifies with his or her career (Metzlaff, 2015).

To study the career identification and organizational identification of apprentices and to describe and detect the potential of social identity theory for VET, it is necessary to consider correlates and predictors of identification such as job satisfaction, career choice, training quality, and trainer competence among adults and in former studies.

*Job satisfaction* is a subjective feeling influenced by personal and situational factors (Ferreira, 2020). It can be described as a person’s attitude and refers to a positive emotional state resulting from the person’s evaluation of his or her own work and work experiences (Wegge and van Dick, 2006). A positive evaluation of an alignment with organizational goals leads individuals to feel satisfied with their job (Lee et al., 2015).

*Career choice* is an important developmental task (Havighurst, 1974) involving processes of vocational socialization (e.g., in internships). As Marcia (1966) emphasizes, the quality of career choice can differ in terms of exploration and commitment. VET aims to prepare learners to act competently in their work environment and to develop an appropriate vocational self-image (Baethge et al., 2007; Kirchknopf and Kögler, 2020).

Participation in a VET program often marks the initial phase of integration into a new social environment and presents a major challenge for young people with the consequences described above. However, organizations can prevent trainee dropout by providing several supportive measures such as onboarding, high quality training, and competent trainers. The *quality of training and trainer competence* have been the focus of scientific and political discourse for many years. Theoretical models postulate connections between the input quality and process quality of the training (i.e., the organization, resources, content, and methods of learning in the work context, and the competence and behavior of trainers) and the output of training (i.e., professional skills, social competence, and professional identity; Klotz et al., 2017).

### 2.2 Empirical research on prerequisites, correlates, and effects of career identification and organizational identification

#### 2.2.1 The importance of career identification and organizational identification in general

The importance of career identification and organizational identification is evident and has been well researched. Identification unfolds its pro-organizational effect by linking the organization with self-concept; therefore, the well-being of the organization - beyond material compensation - also means the well-being of the member. Many studies have shown that identification has a positive influence on several processes that promote interaction between an organization and its members. These include the willingness to do more than required contractually (cf. Van Dick et al., 2006), a reduced probability of resignation (Van Dick et al., 2004), increased creativity (Hirst et al., 2009), and customer orientation (Wieseke et al., 2007). Employees who identify with their chosen career and organization are more satisfied, think less about resigning, are healthier, and have a reduced risk of stress and burnout (Van Dick et al., 2004; Haslam and Van Dick, 2011; van Dick, 2017; Frenzel et al., 2022).

Employees with a high level of identification feel they have support in the organization and can resolve issues together with other members of their group, thus strengthening their resilience (Van Dick and Haslam, 2012). They look at positive aspects of their organization to achieve the best possible self-concept. Positive evaluation and alignment with organizational goals then lead to more satisfaction with their work (Riketta, 2005; Lee et al., 2015).

Employees who identify with their organization are interested in the success of their organization and want to contribute to it. This is because the success of the organization enhances their social identity (Felfe, 2008). Employees devote themselves to tasks outside the formal requirements of their contract, support the organization beyond their duties, and tend to stay in the organization (van Dick et al., 2004; Riketta, 2005).

#### 2.2.2 Career choice as a predictor of identification

Studies of the influence of career choice processes on career identification and organizational identification have been scarce; however, findings of the few studies conducted support the assumption that career choice processes might have an impact on the development of identification. Böhn and Deutscher (2022) found in various qualitative studies that career choice is important for preventing dropout in vocational education and that a good match of the preferred training program/occupation and finally chosen career predicts perseverance in a vocational training program and indicates an attachment to the career and organization at least during the apprenticeship. Choosing an occupation after intensive and systematic career exploration may lead to greater coherence of one’s aims and occupation and to greater job satisfaction (Grotevant et al., 1986; Hirschi, 2008). In-depth exploration leads to more in-depth knowledge in a field and can be fostered by giving adolescents the opportunity to gain relevant experience and perform typical tasks in the occupation. Exploration as the process of searching for relevant and detailed information is linked to identity development, also in the context of career choice and VET enrolment (Kracke and Heckhausen, 2008). We can assume that a well-informed career choice may predict identification with the occupation. Consequently, a study of career identification and organizational identification should explicitly consider whether the trainees’ chosen occupation was his or her first choice, and how this is related to identification.

#### 2.2.3 Training quality and trainer competence as predictors of identification

In a large study of approximately 6,000 trainees in 15 occupations in Germany (Beicht et al., 2009), the perceived quality of training was examined. Findings indicate that for just under a quarter of the trainees the criteria of good training were fulfilled to a high degree. Approximately 50% had a more reserved view (quality criteria mostly fulfilled) on training quality, and just under a quarter (21%) rated the quality of their training as rather poor. A recent meta-analysis has revealed that poor training quality in the form of organization-related factors, in particular learning conditions, working conditions, and work climate predicted dropout (Böhn and Deutscher, 2022).

Findings from studies of the pedagogical ethos of vocational trainers (Forster-Heinzer, 2015; Forster-Heinzer, 2020) indicate that when trainers are engaged, caring, and fair and provide authentic learning opportunities at the workplace, apprentices identify more strongly with the occupation and, particularly, with the organization. The results of multiple regression analyses in a study of more than 500 apprentices in the culinary and automotive fields in Switzerland indicate that both identification with the career (explanation of variance 27.0%) and identification with the organization (explanation of variance 54.4%) were influenced by the trainers’ behavior. The apprentices showed stronger identification when they felt their trainers cared for their vocational and personal development and when they felt their trainers believed in their competence. Moreover, the experience of injustice or unfair treatment led to a decrease in identification with both the organization and occupation.

#### 2.2.4 The relationship between identification and job satisfaction

A positive relationship between identification and job satisfaction has been found in numerous studies and meta-analyses. Two well-known models are discussed in the literature regarding the connection between job satisfaction and identification. The first model assumes that job satisfaction is influenced by identification, while the second assumes identification is influenced by job satisfaction. The models have been tested in various studies (van Dick, 2017). In a survey of employees of a hospital and a university Randsley de Moura et al. (2009) found evidence that the second model was accurate. In contrast, van Dick et al. (2004) found in a similar study that the first model was more strongly reflected in the data. Riketta’s (2005) meta-analysis also confirmed the positive effect of organizational identification on job satisfaction (see also Van Dick et al. (2008) and Gümüs et al. (2012)); however, both directions of effect are plausible when referring to the theoretical bases of job satisfaction, which has a positive effect on employees’ health, leads to more enjoyment at work, and increases self-esteem (Ferreira, 2020). For organizations, job satisfaction means better employee performance, decreased absenteeism, and reduced turnover (Felfe and Six, 2006).

## 3 The present study

### 3.1 Research contribution

Findings of numerous studies of work and organizational psychology indicate that (1) there is a close connection between employees’ career and organizational identification, (2) predictors such as career choice, training quality, and trainer competence might influence identification, and (3) there is a relationship between identification and job satisfaction. In general, employees who identify with their career or organization perform better, are more satisfied, and tend to stay longer in their job or at their organization. However, little investigation has been made into the career identification and organizational identification of apprentices in VET programs, relevant predictors, or outcomes. And in light of the developments described in the introduction (less skilled workers, increasing dropout in VET programs) and the positive effects of identification in other studies it seems worthwhile to investigate identification in VET and to close this research gap.

Results of previous studies suggest that if apprentices receive training in their preferred VET program, the quality of their training is satisfactory, and they perceive their trainers as being competent, they should identify more strongly with their career and organization, which leads to greater job satisfaction. We address and analyze these interrelations in our study. To determine whether previous findings on career identification and organizational identification of employees can be found similarly for the VET sector, apprentices’ career choice and perception of training quality and trainer competence as well as the role of training satisfaction will be explored to understand the extent to which these factors impact or align with the career identification and organizational identification of apprentices.

### 3.2 Research questions

Workplace training in VET programs marks an important transition from school to work for adolescents and young adults, a period during which foundations are built for the development of a vocational identity as well as professional competence. If apprentices receive training in their preferred VET program and they perceive the quality of their training, and their trainer’s competence as being high this should have a significant impact on their learning success, satisfaction with training, career identification, and organizational identification, which we address in this contribution. To determine whether findings concerning the career identification and organizational identification of other employees extend to the VET sector, we explore the following questions:

1. To what degree do apprentices identify with their career and organization and how satisfied are they with their training?
2. How do apprentices perceive the quality of their training and their trainers’ competence?
3. What is the relationship between the apprentices’ perception of training quality and their trainers’ competence, their satisfaction with training, career identification, and organizational identification while considering the apprentices’ choice of preferred VET program?

## 4 Method

### 4.1 Sample and procedure

To answer our research questions, we conducted a cross-sectional study in Germany and Austria in 2020/2021 using an online questionnaire. Because it was a convenience sample, there is no evidence to assume cluster or bias. Of the *N* = 598 apprentices whom we surveyed, 261 were female, 331 were male, and 3 identified as other. The mean age was 19.6 years, with age ranging from 16 to 35. The native language of the apprentices was predominantly German (77%); 23% of the participants spoke a language other than German as their native language. Of the 598 apprentices 46.6% were in a commercial apprenticeship program, 23.8% in a technical apprenticeship, 17.7% were in the retail trade, 1.3% were in the gastronomy sector, and 10.3% were enrolled in nursing professions. Participants were distributed across all 4 years of the apprenticeship programs (1st year: 16.6%; 2nd year: 64.8%; 3rd year: 17.3%; 4th year: 1.2%). Because only a few apprenticeships are designed for a four-year period, the proportion of fourth-year apprentices was low. The size of companies in the sample ranged from small (1–50 employees, 43.6%) over medium (51–250 employees, 13%) to large (more than 250 employees, 38.1%).

### 4.2 Measures and analyses

We conducted the survey using well established Likert scale instruments that showed satisfying to excellent reliability (*α* = 0.687 to 0.930).

#### 4.2.1 Quality of training

To assess the apprentices’ perception of the quality of their training, we used an instrument (ELMA, Rausch, 2012) with five subscales each with very good reliability (task complexity, four items, e.g., “My current tasks are not too challenging,” *α* = 0.768; planning autonomy, two items, e.g., “In my current work, I can organize my tasks myself,” *α* = 0.859; decision autonomy, three items, e.g., “I can choose whether or not I take on a task,” *α* = 0.863; method autonomy, three items, e.g., “In my current work I can choose how I fulfill a task,” *α* = 0.883; task variety, four items, “In my current work I have to master a high variety of tasks,” *α* = 0.916). All items were rated on six-point Likert scales.

#### 4.2.2 Trainer’s competence

We measured the apprentices’ perception of their trainer’s competence using an instrument (Beicht et al., 2009) with two subscales (pedagogical aptitude, five items, e.g., “My instructor can answer difficult questions,” *α* = 0.687; trainer presence, four items, e.g., “There is always an instructor available if I have questions,” *α* = 0.843). The items were rated on six-point Likert scales.

#### 4.2.3 Training satisfaction

We assessed the apprentices’ satisfaction with their training using an instrument (KAFA, Haarhaus, 2015) consisting of five items to be rated on four-point Likert scales (e.g., “All in all, I am satisfied with my training program”). Internal consistency was good (*α* = 0.714).

#### 4.2.4 Career and organizational identification

We measured the apprentices’ identification with their occupation (e.g., “I feel I belong to my professional group”) and the organization (e.g., “I feel I belong to the company I work for”) where they were working and receiving training using an established instrument (Doosje et al., 1995) in a version adapted for use in Germany (Sebastian and Van Dick, 2015). The scales each consisted of four items and showed very good internal consistency (career identification *α* = 0.930; organizational identification *α* = 0.928).

#### 4.2.5 Preferred occupation

We asked the apprentices if the occupation their training was designed to prepare them for was their preferred occupation. The item was scaled dichotomously and considered in the structural equation model; 68% of the participants surveyed stated their chosen occupation was also their preferred occupation.

Addressing the first and second research questions, we focus on the descriptive parameters and relationships between the constructs being measured to determine the extent of the apprentices’ career identification and organizational identification and their views on career choice, training satisfaction, training quality, and trainer competence. To analyze connections among these variables and their direct and indirect effects on career identification and organizational identification we calculated structural equation models. The data was estimated using the maximum likelihood estimator. Missing values were deleted listwise leading to a sample size of *N* = 503 for the SEM analyses. We conducted the analyses using the psych (Revelle, 2022) and lavaan R packages (Rosseel, 2012).

## 5 Results

### 5.1 Descriptive statistics and correlations between the constructs measured

The first research question addressed the organizational identification and career identification of VET apprentices and their perceptions of training quality and trainer competence. On average, the apprentices reported having rather strong identification with their career and with their organization (*M* = 3.16 [occupation] and *M* = 3.19 [organization] on a scale from 1 “do not agree at all” to 4 “completely agree”). The standard deviation amounted to 0.76 (career identification) and 0.80 (organizational identification). Both subconstructs correlated significantly with each other (*r* = 0.507**).

The second research question addressed apprentices’ satisfaction with their training. The values were just below the theoretical scale mean of 2.5 (scale from 1 to 4; *M* = 2.40, SD = 0.41), while the apprentices’ mean perception of training quality was mostly above the theoretical scale mean of 3.5 (scale from 1 to 6), as they rated task variety best (*M* = 4.09, SD = 1.19), followed by the perceived autonomy of task realization (*M* = 3.74, SD = 1.19), decision-making (*M* = 3.71, SD = 1.20), and planning (*M* = 3.49, SD = 1.30). The perceived complexity of tasks averaged at *M* = 2.66 (SD = 1.07) and was slightly lower. The apprentices rated their trainers’ competence as being at a relatively high level (*M* = 4.58, SD = 1.20) and their trainers’ presence as well, averaging at *M* = 4.27 (SD = 1.20).

We calculated bivariate correlations between all measured constructs, which are shown in Table 1 and point to significant relationships between identification and trainers’ competence while task complexity and perceived autonomy in planning did not seem to play a superordinate role in this sample.

**Table 1:** Bivariate correlations between constructs.

	1	2	3	4	5	6	7	8	9	10
1) Training satisfaction	1	0.090*	0.171**	0.186**	0.194**	0.044	0.046	0.097*	0.111**	0.084*
2) Career identity		1	0.507**	0.335**	0.316**	−0.117**	0.134**	0.261**	0.245**	0.386**
3) Organizational identity			1	0.647**	0.611**	−0.099*	0.233**	0.306**	0.322**	0.513**
4) Trainers’ aptitude				1	0.758**	−0.093*	0.238**	0.318**	0.337**	0.513**
5) Trainers’ presence					1	−0.069	0.234**	0.268**	0.307**	0.496**
6) Task complexity						1	0.101*	−0.030	−0.032	−0.382**
7) Autonomy in planning							1	0.525**	0.476**	0.153**
8) Autonomy in decision making								1	0.664**	0.345**
9) Autonomy in task realization									1	0.386**
10) Task variety										1

*= 5% level of significance; **= 1% level of significance; Bold values are significant correlations.

### 5.2 Direct and indirect effects on career identification and organizational identification

**Figure 1:**
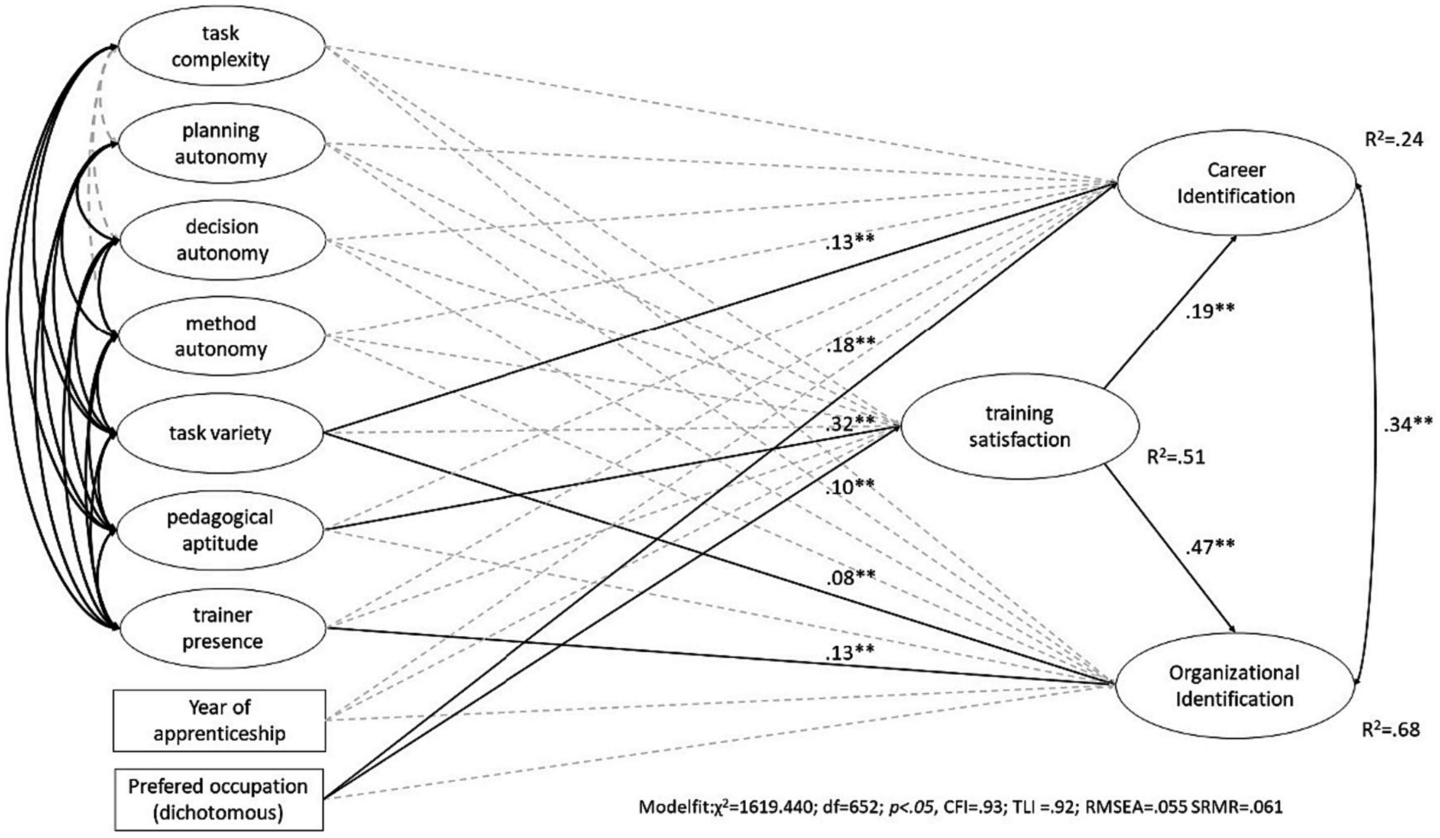
Structure equation model explaining career and organizational identification.

To address the third research question, we analyzed the relationships among training quality, training satisfaction, and identification, and then we extended the model by integrating the year of apprenticeship and whether the current VET program was the preferred one. As a base model, we tested career identification and organizational identification as well as training satisfaction being directly regressed on training quality and trainer competence. This model did not show a satisfying model fit (*χ*2 = 1449.895; df = 584; *p* < 0.05, CFI = 0.94; TLI =0.93; RMSEA = 0.054; SRMR = 0.048) and was further specified. In the second model, the dependent variables career identification and organizational identification were regressed on training quality while being mediated by training satisfaction. The estimated model shows a satisfying fit (*χ*^2^ = 1449.895; df = 584; *p* < 0.05, CFI = 0.94; TLI =0.93; RMSEA = 0.054; SRMR = 0.048). Finally, in line with previous research findings, we extended the second model by the year of apprenticeship and preferred occupation, which had only a slight impact on the model fit and showed significant effects of preferred occupation on career identification (*χ*2 = 1619.440; df = 652; *p* < 0.05, CFI = 0.93; TLI =0.92; RMSEA = 0.055 SRMR = 0.061, see Figure 1). In detail, the results indicate no direct or indirect effects of task complexity or autonomy in planning, decision making, or task realization on trainee satisfaction or identification. However, the variety of tasks experienced directly affected career identification (0.13**) and organizational identification (0.08**), indicating parts of variance which are not mediated by training satisfaction. In terms of trainer presence, we also found a direct effect on organizational identification (0.13**) but not on career identification. Furthermore, the question of whether the training was in the preferred occupation seemed to be relevant for career identification (0.18**). In addition, we found an indirect effect of the preferred occupation on organizational identification via training satisfaction (0.10**), meaning the preferred occupation seems to affect training satisfaction as well as organizational identification and career identification. Another indirect effect via training satisfaction was found for perceived trainer’s aptitude (0.32**). The model shows the relevance of training quality and job satisfaction for identification with the organization where training is taking place, and this explains 68% of the variance. The regression between job satisfaction, training quality, and organizational identification is significant; however, the results indicate that despite a correlation of 0.34 between career identification and organizational identification, only 24% of the variance in career identification can be explained by the model. Lower regression coefficients between career identification, job satisfaction, and training quality underline this finding. The structural equation modeling shows that job satisfaction and training quality clearly impact organizational identification more than career identification. A further specification of the model based on previous findings including the size of the training company and sectors had no further impact on increasing the explanation of variance and therefore was rejected.

## 6 Discussion, limitations, and outlook

The results of this study indicate that apprentices feel a strong identification with their occupation and with the organization where they are doing their training and are mostly satisfied with the quality of their apprenticeships. The structural equation modeling reveals the relevance of career choice, training quality, and job satisfaction for organizational identification and – slightly less - career identification. The learning and working conditions in the organization, in particular the variety of tasks offered to the apprentices and the trainer’s aptitude explain satisfaction with training and career identification; the trainer’s presence and apprentices’ satisfaction with training explain, to some degree, variance in organizational identification.

Comparable findings can also be found in Böhn and Deutscher (2022) and Klotz et al. (2017). The fact that training quality explains more variance in identification with the company than in identification with the occupation is plausible, because - according to social identity theory (Tajfel and Turner, 1986; Mael and Tetrick, 1992; Hogg and Terry, 2001; Egold and van Dick, 2015) – it is easier to identify with smaller groups. Further, a group working in the same organization is usually smaller than a group of individuals working in the same occupation. Moreover, the trainer competence and training quality are more directly related to the company than to the occupational group, and the apprentices spend much more time in the training organization than at school, where they meet apprentices of the same occupation.

Our findings concerning the conditions of VET within the organization indicate that the trainer’s presence and the variety and complexity of tasks offered to apprentices influence their identification with the occupation and with the organization. Furthermore, the trainer’s aptitude has an impact on apprentices’ training satisfaction, which correlates with identification as well. Because the vocational trainer is responsible for offering tasks to the learners, he or she plays a key role in fostering apprentices’ identification and job satisfaction. The results of this study confirm results of Forster-Heinzer insofar as we find a similar explanation of variance in identification with the career (this study: *R*^2^ = 0.24; Forster-Heinzer, 2020: *R*^2^ = 0.027) and identification with the organization (this study: *R*^2^ = 0.68; Forster-Heinzer, 2020: *R*^2^ = 0.054). Maué et al. (2023) identify similar predictors for organizational identification of trainees. In future research investigation could be made into why the variance in organizational identification is better explained than the variance in career identification. For instance, one could assume that workplace characteristics are quite heterogeneous but rather linked to organizations than the occupation itself. Furthermore, it might be fruitful to include additional institutional and individual characteristics in future studies. Thus, for example the quality of career decision making could be measured more precisely by adding variables of the competence of career decision making or occupational exploration to the preferred occupation. Both studies mentioned here (i.e., Maué et al. and Forster-Heinzer) also underline the key role of the trainers’ presence in apprentices’ development of identification and therefore support our findings.

Our study has some limitations that should be mentioned. First, we surveyed a rather heterogeneous sample, not a randomized or representative sample with regard to sectors of vocational education, size of organization, age, and stage in VET. It is possible that our sample is positively selected in terms of satisfaction and identification. Our study only includes less than 20% first year apprentices, whose identification is usually lower and those with low identification are more likely to drop out in the first year. This might explain the comparably high identification among apprentices. Concerning the validity of our results, it is necessary to consider various perspectives on different occupations, which we took into account only generally as VET sectors. Second, our sample was limited to VET in German-speaking contexts. Third, we used a cross-sectional design with one measurement point only and did not control for sampling units in the sense of multilevel models, which might have biased the estimation of standard errors. Although we had intended to take a broad preliminary look at the question of identification during VET, we could not consider all possible side effects with our sample while trying to receive a testable model. Moreover, deeper analyses revealed that there might be slight differences between the apprentices’ perceptions of the relevance of subscales of training quality in Germany and Austria (Heinrichs et al., 2023). In future qualitative studies investigation should be made into how apprentices perceive the learning environment in their organization and how these training conditions relate to social identification.

Finally, we could not determine whether identification was a result or predictor of job satisfaction despite the comparison of model fits indicating training satisfaction was a mediator variable. Literature provides mostly evidence of the effect of identification on satisfaction. Studies in the school context show that identification can be considered an explanatory factor for satisfaction (Van Dick et al., 2004; Gümüs et al., 2012). A study using bootstrapped mediation analyses also provides support for a positive effect of organizational identification on job satisfaction (Karanika-Murray et al., 2015). This should be explored systematically in further studies, for example by integrating prognostic variables such as intention to dropout or intention to remain in a VET program (Findeisen et al., 2022). Thereby, a longitudinal design would be necessary and would offer opportunities to explore patterns, opportunities, and challenges as well as ups and downs of social identification as a driver of satisfaction during apprenticeships.

Our results can be used to generate hypotheses concerning apprentices’ development of identification; however, further analyses would be needed to test them. Additional analyses such as profile analyses could be conducted to explore whether there are groups of apprentices at risk of dropping out or who are likely to stay in the chosen career and organization upon completion of vocational training.

From an educational perspective, understanding the development of identification is essential. Although empirical exploration can be made into what kind of processes, conditions, and challenges might impact apprentices’ social identification and job satisfaction, social identification theory does not adequately model developmental processes. Thus, there is a need for theoretical progress such as comparing and integrating theoretical approaches of identification processes and identity development (Heinrichs et al., 2022).

Overall, this study provides empirical evidence related to the degree of social identification with the occupation and organization experienced by apprentices in the dual VET system in Germany and Austria. Our results offer a basis for future theoretical and empirical research. Further, our results highlight the impact of vocational orientation on the development of identification. They also underline the key role of vocational trainers in VET, who contribute to apprentices’ development of social identification and help prevent dropout. During the dual VET program, the trainers’ engagement with, and care for, apprentices and their ability to provide authentic learning opportunities of varying complexity in workplace settings can contribute to apprentices’ identification with the organization and with the career; however, professional and pedagogical competences of vocational trainers in organizations differ considerably (Krötz and Deutscher, 2021). Therefore, professionalization of trainers in the dual VET system is essential.

## Data availability statement

The datasets presented in this article are not readily available because when asking for consent we told participants that their data will only be used for our study and not shared. Requests to access the datasets should be directed to wuttke@em.uni-frankfurt.de.

## Author contributions

EW has provided substantial contributions to the conception and design of the study, to data acquisition, data cleaning, preliminary analyses, drafting and revising the work. Ensures that questions related to the accuracy or integrity of any part of the work are investigated thoroughly and resolved. KK as the corresponding author was responsible for the empirical modeling process, wrote the empirical part of the paper, and reviewed and revised the entire paper. AJ took part in the empirical modeling process, performed the data analyses, and created the tables and figures. KH has cooperated with EW in conceptualizing the design of this study and was responsible for collecting data in Austria and contributed to the writing of the theoretical part of the paper, wrote main parts of the discussion and, finally, reviewed and contributed to revising the entire paper.

## Footnotes

^1^However, the contract termination rate varies significantly between regions (Lange, 2019, 2020) and training occupations. The rate within the 20 occupations with the highest and lowest contract termination varied in Germany in 2021 between 4.7 and 50.8% (Berufsbildungsbericht, 2023). Similar tendencies are found in Austria and Switzerland.

^2^In Germany, dual VET programs start after secondary school and run parallel mainly to full-time school-based VET and university education. VET plays a central role in the qualification of future professionals and prepares more than one million trainees per year for work in more than 300 recognized professions. Approximately 70% of the qualification time is done in an organization and 30% of the time is spent in vocational schools. Currently, there are 324 different training programs offered in Germany (BIBB, 2021). In-organization training is structured and takes place under real work conditions. Dual VET lasts from two to three and a half years. For both the school-based and organization-based training, legal frameworks are established that should guarantee high quality training. Trainers for the school-based part of VET programs obtain their qualifications in teacher education programs. Education programs for the in-company part are less standardized, but trainer certificates are needed as well.
